# Concomitant motor responses facilitate the acquisition of multiple timing priors beyond upper-limb contexts

**DOI:** 10.1016/j.isci.2026.115051

**Published:** 2026-02-17

**Authors:** Yuma Tanaka, Riku Takaki, Neil W. Roach, Makoto Miyazaki

**Affiliations:** 1 Graduate School of Integrated Science and Technology, Shizuoka University, Hamamatsu 432-8011, Japan; 2 School of Psychology, University of Nottingham, Nottingham NG7 2RD, UK; 3 Faculty of Informatics, Shizuoka University, Hamamatsu 432-8011, Japan

**Keywords:** Behavioral neuroscience, Neuroscience, Sensory neuroscience

## Abstract

The brain acquires prior distributions of target timing to improve performance in timing tasks. In everyday life, multiple events with distinct temporal statistics occur, requiring the acquisition of multiple priors. Previous work has shown that multiple-prior acquisition is facilitated when a concomitant motor response (CMR)—a nondominant hand movement or vocalization—is performed alongside a dominant-hand timing response, selectively for either of the priors. Notably, CMR effects have been observed in reaching movements, but these were specific to upper-limb contexts and absent when a foot movement was used as the CMR. Here, we show that a concomitant foot response also facilitated multiple-prior acquisition in a manual timing task. Therefore, in timing, CMR effects are not restricted to a specific motor effector combination but generalize across various effector combinations. This flexibility, coupled with the multiple motor effectors in the human body, can support adaptability to diverse temporal statistics in real-world environments.

## Highlights

• Concomitant motor responses (CMRs) facilitate acquisition of multiple timing priors
• CMR effects in force-field learning during reaching are upper-limb specific
• A foot CMR facilitates multiple-prior acquisition in this manual timing task
• Flexible CMR effects support adaptation to diverse temporal statistics

## Graphical abstract

**Figure:**
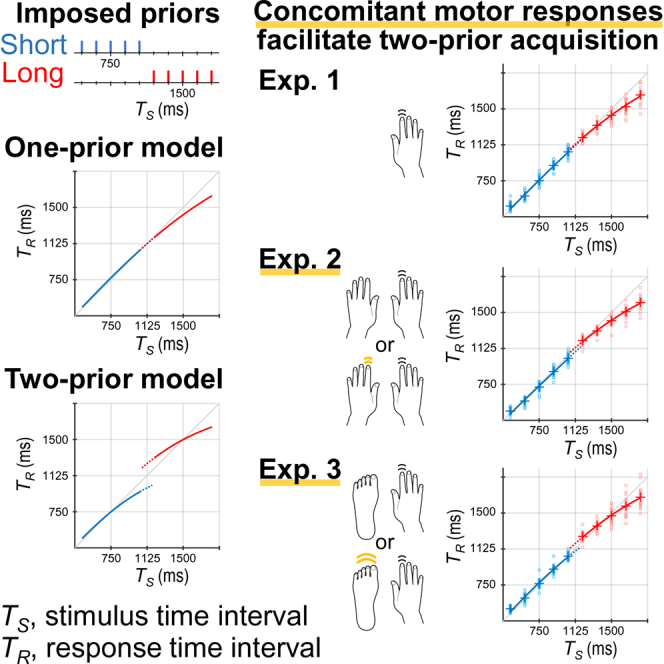

## Introduction

According to the Bayesian estimation model,^1^^,^^2^ the brain can improve its sensorimotor performance by learning the prior distribution of a target and optimally integrating it with current sensory input. Previous studies have demonstrated that human temporal processing aligns with this model,^3^^,^^4^^,^^5^^,^^6^ where target time intervals are selected from a fixed distribution during each task session. However, in everyday tasks, we often face multiple events with different statistical properties (e.g., fast and slow balls in ball sports). To apply Bayesian estimation in natural environments, the brain must concurrently acquire multiple prior distributions.

Recent studies have revealed how behavioral context shapes multiple-prior acquisition in timing tasks. When participants respond only using a manual keypress across different prior distributions (e.g., short and long durations^7^ or intervals^8^^,^^9^), they initially acquire one prior by generalizing over the two distributions (“generalization”). Acquisition of two independent priors is possible in this setup but requires extended training (e.g., approximately 1000 trials^7^). In contrast, when participants use two different types of motor responses (e.g., keypress and vocalization) for the respective priors, they can rapidly acquire two independent priors (“motor specificity”).^7^ Moreover, it has been demonstrated that participants can acquire two independent priors even with a single motor response (keypress), provided that different motor effectors (e.g., right and left hands) are assigned to each prior (“body-part specificity”).^8^

While these results demonstrate that sensorimotor context plays a critical role in shaping multiple-prior acquisition, how this applies to everyday behavior remains unclear. In everyday tasks, it is not always appropriate to switch between motor types or effectors. More recently, Natsume et al. demonstrated that concomitant motor responses (CMRs) facilitate multiple-prior acquisition, even when the same motor type and effector are used throughout.^9^ When participants performed a nondominant hand response concomitantly with a dominant-hand timing response, selectively for either the short or long prior distribution, they effectively acquired two independent priors. Facilitation of multiple-prior acquisition was also observed when vocalization was used as a CMR, suggesting that CMR effects generalize across various combinations of motor responses. Since everyday behaviors often involve complex movements that recruit multiple effectors, strategic variation of CMRs provides a flexible solution to support effective multiple-prior acquisition outside the laboratory.

Currently, it is unclear to what extent the specific type of CMR influences prior acquisition in timing tasks. Notably, evidence from force-field learning in reaching movements suggests that lower-limb responses may not function effectively as a CMR. Nozaki et al.^10^ demonstrated limited transfer of force-field learning within the same arm between bimanual (i.e., with CMR) and unimanual (i.e., without CMR) conditions. This implies that sensorimotor memories differ depending on the presence or absence of a concomitant arm movement. Nozaki et al. also examined whether this effect extended beyond bimanual interactions by employing contralateral ankle flexion as the CMR. The results indicated that sensorimotor memory did not differ with or without a concomitant foot response. Consequently, they concluded that the observed effects were specific to upper-limb contexts.

If force-field learning in reaching and statistical learning in timing share common principles, then CMR effects should not occur when a foot response is used as the CMR in timing tasks. However, unlike reaching—which typically involves the upper limbs—timing plays a role in a wide range of behaviors that engage various effectors, including the voice, upper limbs, and lower limbs.^11^ Therefore, we inferred that the acquisition of multiple priors in timing tasks can be facilitated even when a foot response is used as a CMR. Accordingly, the present study examined whether a concomitant foot response facilitates multiple-prior acquisition in a manual timing task.

## Results

**Figure 1:**
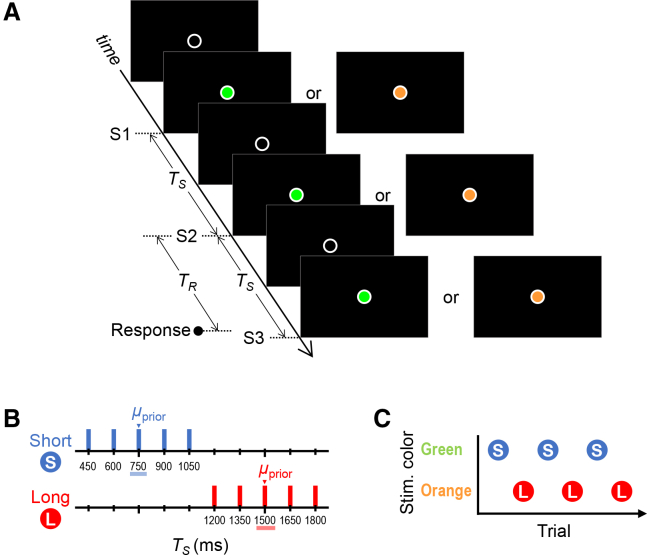
General experimental settings (A) The stimulus sequence and response in each trial. (B) Prior distributions: *T*_*S*_ for each trial was randomly selected from the short or long prior. (C) The short and long priors were assigned to either green or orange stimuli and appeared alternately, trial by trial. *T*_*S*_, stimulus time interval; *T*_*R*_, response time interval; *μ*_prior_, mean of the prior distribution.

Sixty individuals participated in Experiments 1–3 (20 participants per experiment). Participants performed a coincidence timing task (Figure 1A). Three sequential visual stimuli (S1, S2, and S3) were presented at the center of the display. Each sequence of stimuli was colored either green or orange. In each trial, the stimulus time interval (*T*_*S*_) between S1 and S2 was identical to that between S2 and S3. Based on the *T*_*S*_ between S1 and S2, participants attempted to press a button to coincide with the onset of S3. They used their dominant index finger to respond. Each participant completed 640 trials of the timing task. The time interval from the onset of S2 to that of the response was measured as the response time interval (*T*_*R*_).

The *T*_*S*_ was randomly sampled from one of two prior distributions (Figure 1B): a short prior (450–1050 ms; mean [*μ*_prior_] = 750 ms) and a long prior (1200–1800 ms; *μ*_prior_ = 1500 ms). The short and long priors were assigned to either green or orange stimuli (Figure 1C). These prior-color pairings were counterbalanced across participants. Green and orange stimuli were presented in alternation. As a result, short and long priors were also imposed alternately. In our previous studies,^8^^,^^9^ two distinct priors were presented in random order with spatial cues. However, the current study adopted the alternating presentation of two distinct target states with color cues, in accordance with the methods used by Nozaki et al.^10^

**Figure 2:**
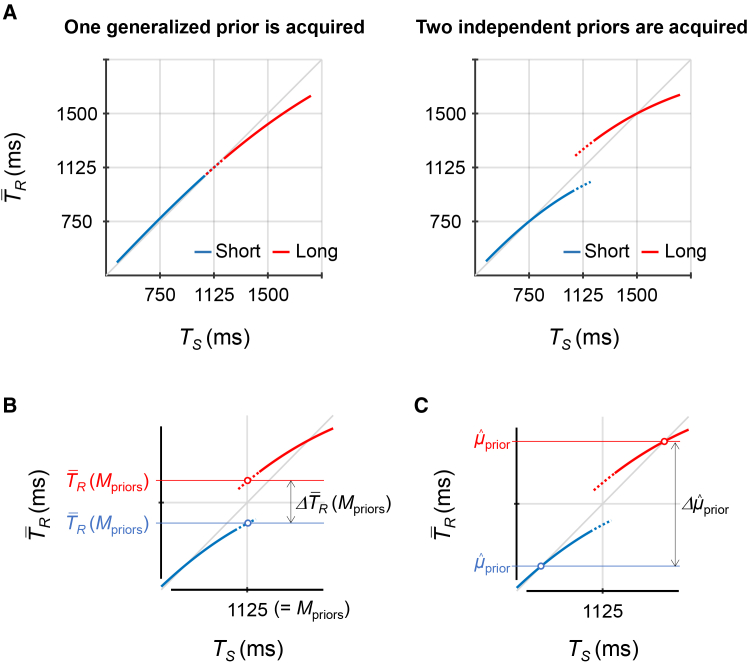
Theoretical predictions and evaluation metrics (A) Predicted ${\bar{T}}_{R}$ as a function of *T*_*S*_ when participants acquire one generalized prior (left) and when participants acquire two independent priors (right). (B) Whether participants acquired two independent priors was evaluated using ${\bar{T}}_{R}\left({Μ}_{\text{priors}}\right)$—${\bar{T}}_{R}$ at *T*_*S*_ = *Μ*_priors_ on the ${\bar{T}}_{R}$ × *T*_*S*_ curves. (C) ${\hat{\mu }}_{\text{prior}}$ can be inferred by the point where the ${\bar{T}}_{R}$ × *T*_*S*_ curves intersect the unity line. ${\bar{T}}_{R}$, mean across trials of *T*_*R*_; *Μ*_priors_, mean over the two prior distributions; ${\hat{\mu }}_{\text{prior}}$, mean of the prior distribution that participants acquired; $\Delta {\bar{T}}_{R}\left({Μ}_{\text{priors}}\right)$, divergence in ${\bar{T}}_{R}\left({Μ}_{\text{priors}}\right)$ between the priors; $\Delta {\hat{\mu }}_{\text{prior}}$, divergence in ${\hat{\mu }}_{\text{prior}}$ between the priors.

### Theoretical predictions

We evaluated how participants acquired short and long priors based on a Bayesian timing estimation model (Equation 1, data analysis in method details). Figure 2 shows the theoretical predictions of ${\bar{T}}_{R}$ (*T*_*R*_ averaged across trials) as a function of *T*_*S*_. If participants acquired one generalized prior, the ${\bar{T}}_{R}$ × *T*_*S*_ curves for both priors would overlap (left panel in Figure 2A). This occurs because timing estimates are biased toward the generalized mean over the two prior distributions (*Μ*_priors_, 1125 ms in this study). In contrast, if participants acquired two independent priors, the ${\bar{T}}_{R}$ × *T*_*S*_ curves would diverge as two separate curves (right panel in Figure 2A). This reflects that timing estimates are biased toward the respective means of the two prior distributions.

To quantitatively evaluate whether participants acquired two independent priors, we used ${\bar{T}}_{R}\left({Μ}_{\text{priors}}\right)$^8^^,^^9^—that is, ${\bar{T}}_{R}$ at *T*_*S*_ = *Μ*_priors_ (Figure 2B; for details, see data analysis in method details). This value accounts for the *μ*_prior_ acquired by participants (${\hat{\mu }}_{\text{prior}}$) both theoretically and empirically.^8^ If participants acquired one generalized prior, the ${\bar{T}}_{R}\left({Μ}_{\text{priors}}\right)$ values would not differ between the two priors. If participants acquired two independent priors, the ${\bar{T}}_{R}\left({Μ}_{\text{priors}}\right)$ value would be greater for the long prior than for the short prior.

In theory, ${\hat{\mu }}_{\text{prior}}$ can be directly inferred from the point where the ${\bar{T}}_{R}$ × *T*_*S*_ curve intersects the unity line (Figure 2C). However, in practice, the intersection points are highly sensitive to individual variations in response, which limits their utility for individual analyses (for details, see data analysis in method details). To address this, we calculated ${\hat{\mu }}_{\text{prior}}$ using the grand-averaged ${\bar{T}}_{R}$ values (i.e., means across participants). We then conducted permutation analyses^9^ to statistically compare the ${\hat{\mu }}_{\text{prior}}$ values between the control experiment without CMR (Experiment 1) and the experiments with CMR (Experiments 2 and 3). In addition, we evaluated whether the two-prior model provided a better fit to participants’ responses than the one-prior model using Akaike weights^12^ (see quantification and statistical analysis).

### Experiment 1: Timing without CMR (control)

**Figure 3:**
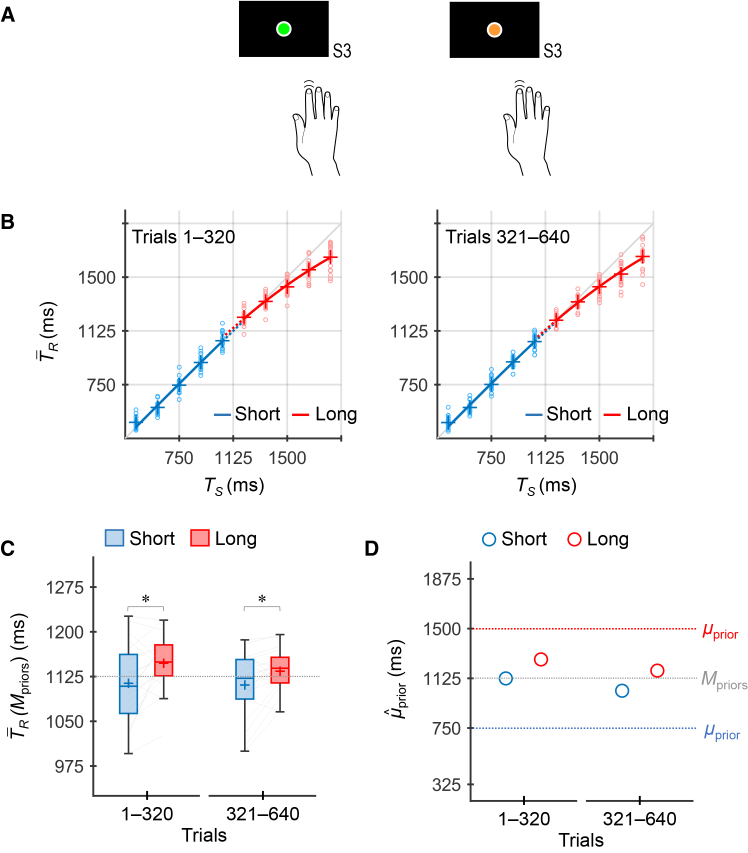
Experiment 1 (control) (A) Participants (*n* = 20) performed the timing task using only the dominant hand regardless of the stimulus colors (i.e., priors). (B) ${\bar{T}}_{R}$ values across participants as a function of *T*_*S*_, calculated per 320 trials. The blue and red markers represent the values for the short and long priors, respectively. The small circle markers represent the individual values. The plus markers represent the grand-averaged ${\bar{T}}_{R}$ values (mean across participants). The blue and red lines represent the curves fitted to the grand-averaged ${\bar{T}}_{R}$ values for the short and long priors, respectively. (C) ${\bar{T}}_{R}\left({Μ}_{\text{priors}}\right)$ values across participants, calculated per 320 trials. The blue and red boxes represent the values for the short and long priors, respectively. The upper and lower limits of each box represent the third and first quartiles (Q3 and Q1), respectively. The upper and lower whiskers indicate the maximum and minimum values within Q3 + 1.5 × interquartile range (IQR) and Q1 − 1.5 × IQR, respectively. The plus sign in each box indicates the mean across participants. The left and right ends of each thin gray line indicate the individual values for the short and long priors, respectively. The asterisks denote the statistically significant results of post-hoc simple effect analyses for prior (trials 1–320: *F*_1, 19_ = 8.37, *p* = 0.0093, *η*_*p*_^2^ = 0.31; trials 321–640: *F*_1, 19_ = 7.22, *p* = 0.015, *η*_*p*_^2^ = 0.28) with Bonferroni correction (∗*p* < 0.05/2), based on a two-way repeated-measures analysis of variance (ANOVA) with factors of prior and trial bin. Although neither the main effect of trial bin nor the interaction between prior and trial bin was significant, the results of simple effect analyses are shown for consistency with Figure 4C (Experiment 2) and Figure 5C (Experiment 3). (D) ${\hat{\mu }}_{\text{prior}}$ values inferred using the grand-averaged ${\bar{T}}_{R}$ values per 320 trials. The blue and red open circles represent the values for the short and long priors, respectively.

Experiment 1 was conducted to characterize baseline performance in the absence of any CMR. Participants performed the timing task using only their dominant index finger regardless of the stimulus colors (i.e., priors) (Figure 3A).

Figure 3B shows the ${\bar{T}}_{R}$ values across participants as a function of *T*_*S*_. The ${\bar{T}}_{R}$ × *T*_*S*_ curves for the short and long priors were approximately overlapping, with small separations in both trial bins (trials 1–320 and 321–640). As shown in Figure 3C, the ${\bar{T}}_{R}\left({Μ}_{\text{priors}}\right)$ values for the long prior were higher than those for the short prior in both trials 1–320 and 321–640. A repeated-measures two-way analysis of variance (ANOVA) with factors of prior and trial bin revealed a significant main effect of prior (*F*_1, 19_ = 9.78, *p* = 0.0055, *η*_*p*_^2^ = 0.34) but no significant effect of trial bin (*F*_1, 19_ = 1.56, *p* = 0.23, *η*_*p*_^2^ = 0.076) or interaction (*F*_1, 19_ = 1.24, *p* = 0.28, *η*_*p*_^2^ = 0.061). The ${\hat{\mu }}_{\text{prior}}$ values followed a similar pattern to the ${\bar{T}}_{R}\left({Μ}_{\text{priors}}\right)$ values (Figure 3D).

Although these results suggest that participants acquired two independent priors, the divergence between them was subtle. Moreover, model comparisons did not consistently support the two-prior acquisition. While the Akaike weights favored the two-prior model over the one-prior model in trials 1–320 (one/two: 0.15/0.85), the reverse was true in trials 321–640 (one/two: 0.67/0.33).

### Experiment 2: Timing with a CMR by the nondominant hand

**Figure 4:**
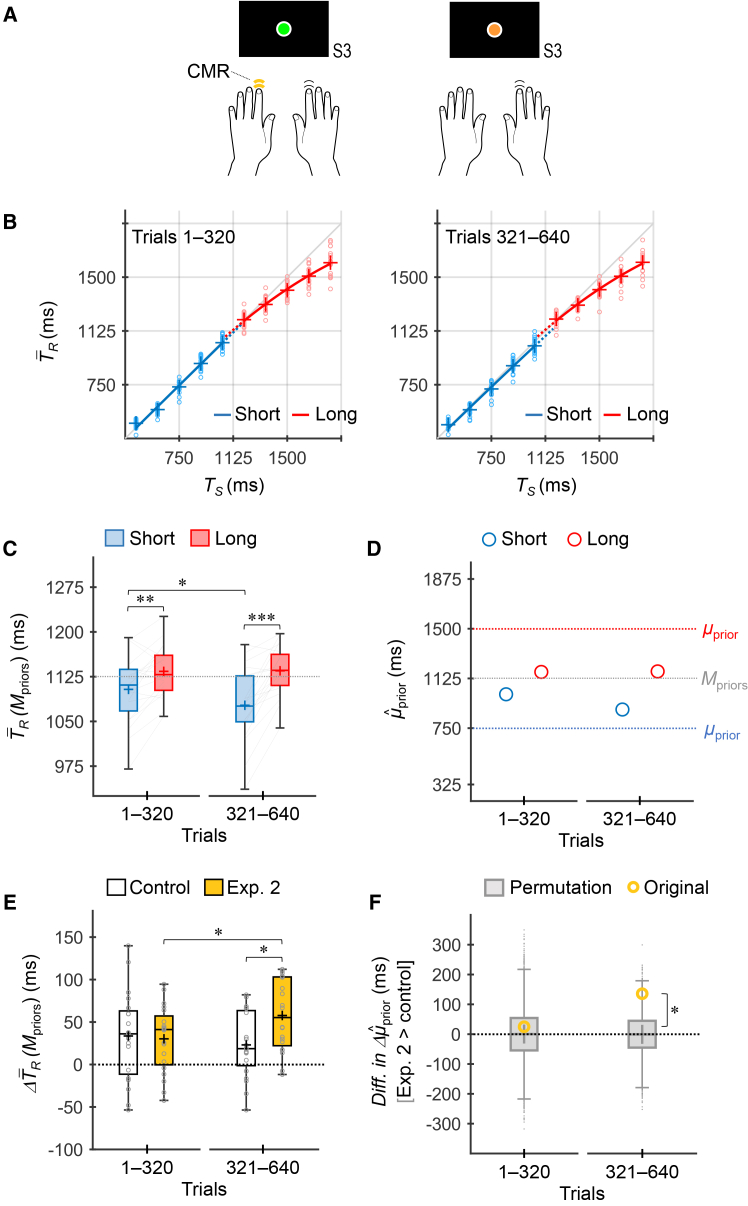
Experiment 2 (CMR by the nondominant hand) (A) Participants (*n* = 20) selectively added a nondominant hand response concomitantly with the dominant-hand timing response according to the stimulus colors (i.e., priors). (B) ${\bar{T}}_{R}$ values across participants as a function of *T*_*S*_. Marker, line, and box representations in B–D are the same as those in Figure 3. (C) ${\bar{T}}_{R}\left({Μ}_{\text{priors}}\right)$ values across participants. The asterisks denote the statistically significant results of post-hoc simple effect analyses for prior (trials 1–320: *F*_1, 19_ = 12.10, *p* = 0.0025, *η*_*p*_^2^ = 0.39; trials 321–640: *F*_1, 19_ = 37.79, *p* = 6.6 × 10^−6^, *η*_*p*_^2^ = 0.67) and trial bin (short: *F*_1, 19_ = 9.24, *p* = 0.0068, *η*_*p*_^2^ = 0.33; long: *F*_1, 19_ = 0.013, *p* = 0.91, *η*_*p*_^2^ = 0.0007) with Bonferroni correction (∗*p* < 0.05/2, ∗∗*p* < 0.01/2, ∗∗∗*p* < 0.001/2), based on a two-way repeated-measures ANOVA. (D) ${\hat{\mu }}_{\text{prior}}$ values inferred using the grand-averaged ${\bar{T}}_{R}$ values. (E) $\Delta {\bar{T}}_{R}\left({Μ}_{\text{priors}}\right)$ values across participants for the control experiment (open boxes) and Experiment 2 (deep-yellow-filled boxes). The upper and lower limits of each box represent Q3 and Q1, respectively. The upper and lower whiskers indicate the maximum and minimum values within Q3 + 1.5 × IQR and Q1 − 1.5 × IQR, respectively. The plus marker in each box indicates the mean across participants. The small gray circles represent the individual values. The asterisks denote the statistically significant results of post-hoc simple effect analyses for experiment (trials 1–320: *F*_1, 38_ = 0.054, *p* = 0.82, *η*_*p*_^2^ = 0.0014; trials 321–640: *F*_1, 38_ = 7.39, *p* = 0.0098, *η*_*p*_^2^ = 0.16) and trial bin (control: *F*_1, 19_ = 1.24, *p* = 0.28, *η*_*p*_^2^ = 0.061; Exp. 2: *F*_1, 19_ = 9.75, *p* = 0.0056, *η*_*p*_^2^ = 0.34) with Bonferroni correction (∗*p* < 0.05/2), based on a mixed-design two-way ANOVA with one between-participant factor of experiment and one within-participant factor of trial bin. (F) Differences in $\Delta {\hat{\mu }}_{\text{prior}}$ between the experiments (Exp. 2 > control) obtained from the original data (open deep-yellow circles) and permutation data (gray-filled boxes). For the permutation data, the upper and lower limits of each box represent Q3 and Q1, respectively. The upper and lower whiskers indicate the maximum and minimum values within Q3 + 1.5 × IQR and Q1 − 1.5 × IQR, respectively. The plus sign in each box indicates the mean for the permutation data. The asterisk denotes the statistically significant result of permutation analyses (trials 1–320: permutation *p* = 0.38; trials 321–640: permutation *p* = 0.019) with Bonferroni correction (∗ permutation *p* < 0.05/2). CMR, concomitant motor response.

Experiment 2 was conducted to confirm whether the facilitation of multiple-prior acquisition by the concomitant nondominant-hand response^9^ was reproduced under the current experimental conditions. Participants were instructed to selectively press an additional button with their nondominant index finger, simultaneously with the timing response made by their dominant hand, based on the stimulus colors (i.e., priors) (Figure 4A).

As shown in Figure 4B, the ${\bar{T}}_{R}$ × *T*_*S*_ curves for the two priors nearly overlapped, with a slight difference, during trials 1–320. The curves began to diverge during trials 321–640. The ${\bar{T}}_{R}\left({Μ}_{\text{priors}}\right)$ values for the long prior were greater than those for the short prior in both trials 1–320 and 321–640 (Figure 4C). A repeated-measures two-way ANOVA revealed a significant main effect of prior (*F*_1, 19_ = 30.96, *p* = 2.3 × 10^−5^, *η*_*p*_^2^ = 0.62) and a significant interaction (*F*_1, 19_ = 9.75, *p* = 0.0056, *η*_*p*_^2^ = 0.34), but not a significant effect of trial bin (*F*_1, 19_ = 3.28, *p* = 0.086, *η*_*p*_^2^ = 0.15). Simple-effect analyses showed that the effects of prior were significant in trials 1–320 (*F*_1, 19_ = 12.10, *p* = 0.0025 < 0.05/2, Bonferroni corrected, *η*_*p*_^2^ = 0.39) and 321–640 (*F*_1, 19_ = 37.79, *p* = 6.6 × 10^−6^ < 0.05/2, *η*_*p*_^2^ = 0.67). For trial bin, the simple effect was significant for the short prior (*F*_1, 19_ = 9.24, *p* = 0.0068 < 0.05/2, *η*_*p*_^2^ = 0.33) but not for the long prior (*F*_1, 19_ = 0.013, *p* = 0.91, *η*_*p*_^2^ = 0.0007). The ${\hat{\mu }}_{\text{prior}}$ values were consistent with the ${\bar{T}}_{R}\left({Μ}_{\text{priors}}\right)$ values (Figure 4D). These results suggest that the short and long priors were concurrently acquired. In addition, the acquisition of the short prior was further developed in the latter half of the trials, whereas that of the long prior remained stable; this was also observed in previous studies.^8^^,^^9^

The Akaike weights supported the acquisition of two independent priors in trials 321–640 (one/two: 0.02/0.98), although this was not validated in trials 1–320 (one/two: 0.59/0.41).

Figure 4E shows the divergence in ${\bar{T}}_{R}\left({Μ}_{\text{priors}}\right)$ between the two priors [long > short, $\Delta {\bar{T}}_{R}\left({Μ}_{\text{priors}}\right)$, Figure 2B] for the control experiment (Experiment 1) and Experiment 2. A mixed-design two-way ANOVA with one between-participant factor of experiment and one within-participant factor of trial bin revealed a significant interaction (*F*_1, 38_ = 8.67, *p* = 0.0055, *η*_*p*_^2^ = 0.19), although the main effects of experiment (*F*_1, 38_ = 1.69, *p* = 0.20, *η*_*p*_^2^ = 0.043) and trial bin (*F*_1, 38_ = 1.73, *p* = 0.20, *η*_*p*_^2^ = 0.044) were not significant. The simple effect analyses showed that the effect of experiment was not significant in trials 1–320 (*F*_1, 38_ = 0.054, *p* = 0.82, *η*_*p*_^2^ = 0.0014) but was significant in trials 321–640 (*F*_1, 38_ = 7.39, *p* = 0.0098 < 0.05/2, *η*_*p*_^2^ = 0.16). Thus, the $\Delta {\bar{T}}_{R}\left({Μ}_{\text{priors}}\right)$ values did not differ between the experiments in trials 1–320, whereas in trials 321–640, the value for Experiment 2 was significantly greater than that for the control experiment. For trial bin, the simple effect was not significant in the control experiment (*F*_1, 19_ = 1.24, *p* = 0.28, *η*_*p*_^2^ = 0.061) but was significant in Experiment 2 (*F*_1, 19_ = 9.75, *p* = 0.0056 < 0.05/2, *η*_*p*_^2^ = 0.34), indicating that the $\Delta {\bar{T}}_{R}\left({Μ}_{\text{priors}}\right)$ value increased in the latter half of the trials in Experiment 2, whereas it remained stable in the control experiment.

Figure 4F shows the difference in $\Delta {\hat{\mu }}_{\text{prior}}$ between the experiments (Experiment 2 > control) obtained from the original and permutation data. Here, $\Delta {\hat{\mu }}_{\text{prior}}$ represents the divergence in ${\hat{\mu }}_{\text{prior}}$ between the two priors (long > short, Figure 2C). The permutation data (10000 sets) were generated by randomly permuting the ${\bar{T}}_{R}$ × *T*_*S*_ sequences of each participant between the experiments (for details, see quantification and statistical analysis). In trials 321–640, the difference in $\Delta {\hat{\mu }}_{\text{prior}}$ for the original data was significantly greater than those obtained from the permutation data (permutation *p* = 0.019 < 0.05/2), although this was not significant in trials 1–320 (permutation *p* = 0.38). The results further indicate that CMR increased the divergence between the acquired priors in the latter half of the trials.

Thus, these comparisons between experiments demonstrate that the nondominant-hand CMR facilitated the acquisition of two independent priors, with the effect emerging after a few hundred trials of learning.

### Experiment 3: Timing with a CMR by the contralateral foot

**Figure 5:**
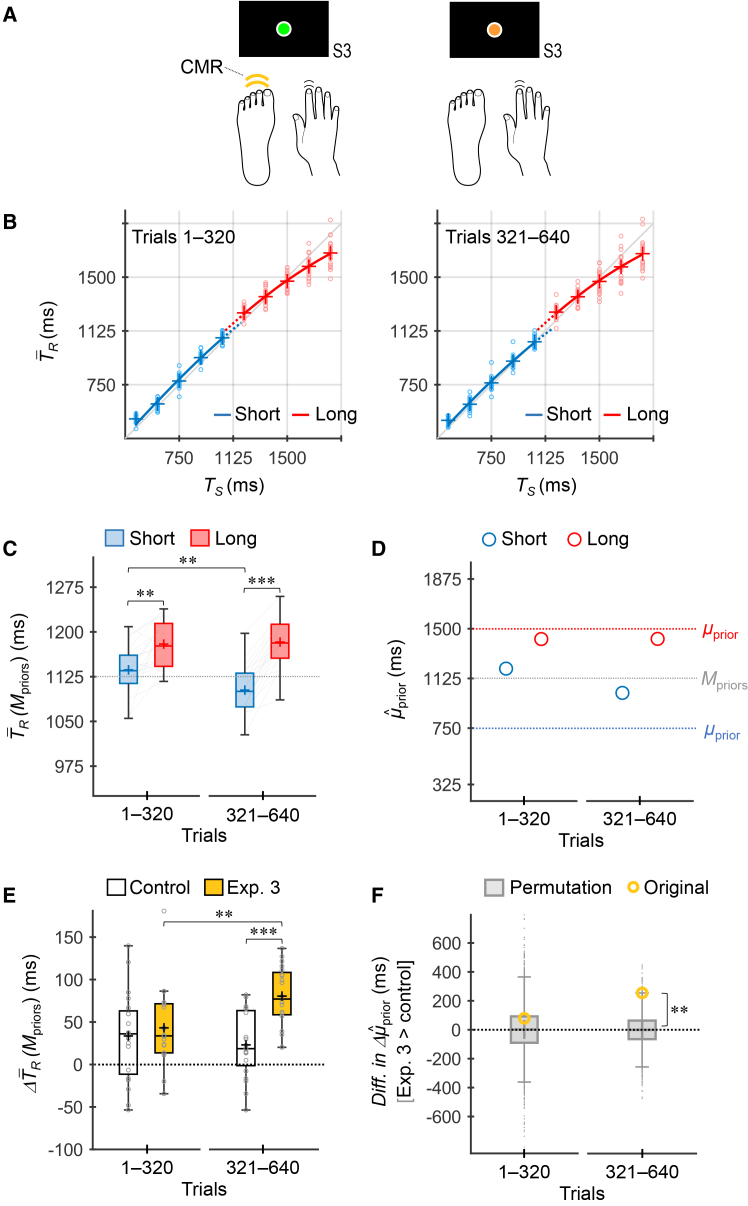
Experiment 3 (CMR by the contralateral foot) (A) Participants (*n* = 20) selectively added a contralateral foot response concomitantly with the dominant-hand timing response according to the stimulus colors (i.e., priors). (B) ${\bar{T}}_{R}$ values across participants as a function of *T*_*S*_. Marker, line, and box representations are the same as those in Figures 3 and 4. (C) ${\bar{T}}_{R}\left({Μ}_{\text{priors}}\right)$ values across participants. The asterisks denote the statistically significant results of post-hoc simple effect analyses for prior (trials 1–320: *F*_1, 19_ = 17.49, *p* = 0.00050, *η*_*p*_^2^ = 0.48; trials 321–640: *F*_1, 19_ = 104.70, *p* = 3.6 × 10^−9^, *η*_*p*_^2^ = 0.85) and trial bin (short: *F*_1, 19_ = 15.03, *p* = 0.0010, *η*_*p*_^2^ = 0.44; long: *F*_1, 19_ = 0.21, *p* = 0.65, *η*_*p*_^2^ = 0.011) with Bonferroni correction (∗∗*p* < 0.01/2, ∗∗∗*p* < 0.001/2), based on a two-way repeated-measures ANOVA. (D) ${\hat{\mu }}_{\text{prior}}$ values inferred using the grand-averaged ${\bar{T}}_{R}$ values. (E) $\Delta {\bar{T}}_{R}\left({Μ}_{\text{priors}}\right)$ values across participants for the control experiment and Experiment 3. The asterisks denote the statistically significant results of post-hoc simple effect analyses for experiment (trials 1–320: *F*_1, 38_ = 0.38, *p* = 0.54, *η*_*p*_^2^ = 0.0099; trials 321–640: *F*_1, 38_ = 24.26, *p* = 1.7 × 10^−5^, *η*_*p*_^2^ = 0.39) and trial bin (control: *F*_1, 19_ = 1.24, *p* = 0.28, *η*_*p*_^2^ = 0.061; Exp. 3: *F*_1, 19_ = 13.68, *p* = 0.0015, *η*_*p*_^2^ = 0.42) with Bonferroni correction (∗∗*p* < 0.01/2, ∗∗∗*p* < 0.001/2), based on a mixed-design two-way ANOVA. (F) Differences in $\Delta {\hat{\mu }}_{\text{prior}}$ between the experiments (Exp. 3 > control) obtained from the original data and permutation data. The asterisks denote the statistically significant result of permutation analyses (trials 1–320: permutation *p* = 0.28; trials 321–640: permutation *p* = 0.0041) with Bonferroni correction (∗∗ permutation *p* < 0.01/2).

In Experiment 3, participants were instructed to selectively press a footswitch with their contralateral forefoot, simultaneously with the timing response made by their dominant hand, based on the stimulus colors (i.e., priors) (Figure 5A).

The ${\bar{T}}_{R}$ × *T*_*S*_ curves for the short and long priors diverged during trials 1–320, and this divergence increased further in trials 321–640 (Figure 5B). The ${\bar{T}}_{R}\left({Μ}_{\text{priors}}\right)$ values for the long prior were greater than those for the short prior in both trials 1–320 and 321–640 (Figure 5C). A repeated-measures two-way ANOVA revealed a significant main effect of prior (*F*_1, 19_ = 65.07, *p* = 1.5 × 10^−7^, *η*_*p*_^2^ = 0.77) and trial bin (*F*_1, 19_ = 5.88, *p* = 0.025, *η*_*p*_^2^ = 0.24), as well as a significant interaction (*F*_1, 19_ = 13.68, *p* = 0.0015, *η*_*p*_^2^ = 0.42). Simple-effect analyses showed that the effects of prior were significant in trials 1–320 (*F*_1, 19_ = 17.49, *p* = 0.00050 < 0.05/2, *η*_*p*_^2^ = 0.48) and 321–640 (F_1, 19_ = 104.70, *p* = 3.6 × 10^−9^ < 0.05/2, *η*_*p*_^2^ = 0.85). For trial bin, the simple effect was significant for the short prior (*F*_1, 19_ = 15.03, *p* = 0.0010 < 0.05/2, *η*_*p*_^2^ = 0.44) but not for the long prior (*F*_1, 19_ = 0.21, *p* = 0.65, *η*_*p*_^2^ = 0.011). Thus, ${\bar{T}}_{R}\left({Μ}_{\text{priors}}\right)$ values were consistent with those in Experiment 2. The ${\hat{\mu }}_{\text{prior}}$ values showed a similar pattern to the ${\bar{T}}_{R}\left({Μ}_{\text{priors}}\right)$ values (Figure 5D).

The Akaike weights were higher for the two-prior model than for the one-prior model in both trials 1–320 (one/two: 0.27/0.73) and 321–640 (one/two: 0.002/0.998), supporting the acquisition of two independent priors.

Figure 5E shows the $\Delta {\bar{T}}_{R}\left({Μ}_{\text{priors}}\right)$ values for the control experiment and Experiment 3. A mixed-design two-way ANOVA revealed a significant main effect of experiment (*F*_1, 38_ = 7.96, *p* = 0.0076, *η*_*p*_^2^ = 0.17) and a significant interaction (*F*_1, 38_ = 11.99, *p* = 0.0013, *η*_*p*_^2^ = 0.24), but the main effect of trial bin was not significant (*F*_1, 38_ = 3.77, *p* = 0.060, *η*_*p*_^2^ = 0.090). The simple-effect analyses showed that the effect of experiment was not significant in trials 1–320 (*F*_1, 38_ = 0.38, *p* = 0.54, *η*_*p*_^2^ = 0.0099) but was significant in 321–640 (*F*_1, 38_ = 24.26, *p* = 1.7 × 10^−5^ < 0.05/2, *η*_*p*_^2^ = 0.39). For trial bin, the simple effect was not significant in the control experiment (*F*_1, 19_ = 1.24, *p* = 0.28, *η*_*p*_^2^ = 0.061) but was significant in Experiment 3 (*F*_1, 19_ = 13.68, *p* = 0.0015 < 0.05/2, *η*_*p*_^2^ = 0.42). These results were consistent with those in Experiment 2.

Thus, Experiment 3 yielded similar results to Experiment 2. In addition, the effect sizes of experiment were larger in Experiment 3 (e.g., *η*_*p*_^2^ = 0.39 in trials 321–640) than in Experiment 2 (*η*_*p*_^2^ = 0.16 in trials 321–640). However, these differences were not statistically significant in the direct comparison between Experiments 2 and 3. A mixed-design two-way ANOVA on the $\Delta {\bar{T}}_{R}\left({Μ}_{\text{priors}}\right)$ values did not reveal a significant main effect of experiment (*F*_1, 38_ = 2.63, *p* = 0.11, *η*_*p*_^2^ = 0.065) or interaction (*F*_1, 38_ = 0.54, *p* = 0.47, *η*_*p*_^2^ = 0.014). In contrast, the effect of trial bin was significant (*F*_1, 38_ = 23.42, *p* = 2.2 × 10^−5^, *η*_*p*_^2^ = 0.38), suggesting that the rates of two-prior acquisition also did not differ between Experiments 2 and 3.

Figure 5F shows the results of the permutation analyses on the difference in $\Delta {\hat{\mu }}_{\text{prior}}$ between the experiments (Experiment 3 > control). In trials 321–640, the difference in $\Delta {\hat{\mu }}_{\text{prior}}$ for the original data was significantly greater than those obtained from the permutation data (permutation *p* = 0.0041 < 0.05/2), whereas this was not significant in trials 1–320 (permutation *p* = 0.28).

Therefore, the foot CMR also facilitated the acquisition of two independent priors, with the effect emerging after a few hundred trials of learning.

## Discussion

The divergence between the two acquired priors became evident after a few hundred trials of learning when a CMR by the nondominant hand (Experiment 2) or the contralateral foot (Experiment 3) was selectively assigned to one of the priors. These results indicate that, in timing, CMRs facilitate multiple-prior acquisition, extending beyond upper-limb contexts to include combinations involving both upper and lower limbs. Furthermore, Natsume et al.^9^ also observed multiple-prior acquisition when a concomitant vocal response was employed during a timing task. These findings suggest that CMR effects are not confined to a specific combination of motor effectors but generalize across various combinations in timing. This flexibility, in combination with the diverse motor effectors of the human body, may support greater adaptability to diverse timing statistics encountered in real-world contexts.

Our current finding on the effectiveness of the concomitant foot response contrasts with the results of a previous study on force-field learning during reaching movements. Nozaki et al.^10^ reported that a concomitant foot movement had minimal influence on manual force-field learning, suggesting that the CMR effect might be limited to upper-limb contexts. A likely explanation for this discrepancy lies in the nature of the tasks—timing versus reaching—although other methodological differences exist between the studies. Reaching is typically a motor pattern carried out by the upper limbs. The lower-limb-independent memories may reflect a neural strategy to stabilize reaching movements across varying lower-limb contexts. If motor memories for reaching were shaped by concurrent lower-limb movements, the consistency of reaching could be unnecessarily disrupted. In contrast, timing is a sensorimotor function embedded across a wide range of behaviors and motor effectors.^11^ Moreover, timing plays a crucial role in coordinating multiple body parts to improve motor performance in daily whole-body behaviors, such as pitching.^13^ For effective timing, the brain requires flexible sensorimotor memories that adapt to diverse body contexts, rather than memories constrained to a specific body context.

Additionally, differences in the time course of multiple-prior acquisition appear across studies using timing tasks. In the present study, divergence between the two acquired priors became evident in the latter half of the trials. In contrast, our previous study,^9^ which involved a concomitant nondominant hand response similar to that in Experiment 2, demonstrated more rapid acquisition of two independent priors, with prior divergence evident in the first half of the trials. Two differences in the experimental settings potentially affect the speed of prior acquisition between the current and previous studies: (1) the visual features of the target stimuli (color versus spatial location) and (2) the order in which the two priors appeared (alternating versus random).

For the visual features of the target stimuli, color (green/orange) was used in the current study. In contrast, spatial location (right/left) was used in the previous study,^9^ in which participants executed the concomitant nondominant hand response (i.e., responded with both hands) when the stimuli appeared on both sides, but responded only with the dominant hand when the stimuli appeared on one side (dominant-hand side). Notably, in unimanual reaching tasks, color cues failed to support the learning of two independent force fields, whereas spatial cues based on the relationship between movements and visual feedback enabled such learning.^14^ A similar contrast was observed in the statistical learning of two opposing orders in tactile temporal order judgment tasks.^15^ Likewise, spatial cues tied to motor responses might further assist the acquisition of two independent priors in the previous timing task.^9^ Alternatively, nonecological associations of colors with motor responses may impose additional cognitive load, drawing on neural resources and thereby slowing the acquisition of priors in the current timing task.

Regarding the order of the priors, the short and long priors were presented in alternating order in the current study, whereas they appeared in random order in the previous study.^9^ A previous study using unimanual reaching tasks^16^ showed that two opposing force fields were learned more effectively when presented in a random order than in an alternating order. Similarly, randomizing the order of priors may have assisted the acquisition of two independent priors in the previous timing study.^9^ In that study, participants had to discriminate targets and select responses on each trial, and this cognitive effort may have aided learning of the two independent priors associated with those targets. In other words, if the random-order effect holds, the current results suggest that CMRs enable the acquisition of multiple timing priors without such cognitive effort, albeit requiring more learning trials.

At present, the neural basis for the interaction between motor contexts and prior acquisition remains unclear. In previous timing studies, the supplementary motor area (SMA) has been proposed to be involved in the concurrent learning of multiple priors.^7^^,^^8^^,^^9^ Roach et al. (2017)^7^ proposed that the SMA plays a role in motor specificity, as SMA neurons show both time-interval tuning^17^ and action selectivity.^18^ Dorsal frontal areas, including the SMA, exhibit neuronal activity reflecting Bayesian timing responses,^19^ supporting the hypothesis. Based on the SMA hypothesis, Matsumura et al. (2024)^8^ planned their experiments because the SMA has somatotopy.^20^^,^^21^ Natsume et al. (2025)^9^ designed their experiments based on previous reports of SMA neurons selectively activated during either unimanual movements (i.e., without CMR) or bimanual movements (i.e., with CMR).^22^^,^^23^ Consequently, they found multiple-prior acquisition based on body-part specificity^8^ and CMRs,^9^ as predicted by the SMA hypothesis. Notably, similar brain networks including the SMA are engaged in both bimanual and hand-foot temporal coordination,^24^ consistent with the SMA hypothesis and the current results.

The current finding—CMR effects are not confined to a specific combination of effectors—may suggest that effector-independent (common) neural processes^25^^,^^26^ are involved in CMR effects in timing behavior. Based on the above discussion, the SMA may contribute to this process in timing. However, neuroimaging studies on interlimb temporal coordination suggest that effector-specific processes^27^^,^^28^^,^^29^ are also involved in hand-foot temporal coordination. In bimanual tasks, interlimb-specific activity was observed in no regions^30^ or in the SMA extending to the cingulate motor area (CMA)^31^ during in-phase movements, and in a broader set of regions, including the SMA^30^^,^^31^ extending to the CMA^31^ and the premotor cortex (PMC),^30^^,^^31^ during anti-phase movements. By contrast, hand-foot tasks consistently induced interlimb-specific activation in multiple regions—the SMA, CMA, PMC, cerebellum, and primary sensorimotor cortex (M1/S1)—during both in- and anti-phase movements,^32^ with enhanced SMA activity during anti-phase movements.^32^^,^^33^

Thus, hand-foot temporal coordination likely not only shares neural bases with bimanual coordination (e.g., SMA, PMC) but also recruits these regions more strongly and engages additional regions (e.g., cerebellum, M1/S1). Notably, neurons selectively activated during either unimanual or bimanual movements were also observed in the PMC.^22^^,^^23^ A theoretical study demonstrated that the cerebellum can learn the prior distribution of time intervals.^34^ In addition, given that bimanual-specific activation patterns have been observed in M1,^35^^,^^36^ hand-foot-specific activity in M1/S1^32^ might reflect enhanced interlimb-specific M1 activity during hand-foot temporal coordination. Besides the SMA, these regions may also contribute, either commonly or specifically, to CMR effects when concomitant foot responses are used.

The present findings have implications for daily sensorimotor behaviors, such as those seen in sports.^37^^,^^38^^,^^39^^,^^40^^,^^41^ For example, baseball batters often swing a bat while performing a leg kick. If batters selectively use a leg kick depending on ball speed, the timing of their bat swing may be optimized for each speed. Moreover, by identifying the shared and distinct effects of CMRs on reaching and timing in future studies, we can further expand insights into daily sensorimotor behavior. In reaching tasks, for instance, participants were able to learn two opposing force fields based on differences in the movements performed before (lead-in)^42^^,^^43^ or after (follow-through)^44^^,^^45^ the main reaching movement within the same hand. Subsequently, Gippert et al. demonstrated that lead-in movements made with the left hand enabled the right hand to learn two opposing force fields.^46^ Accordingly, in timing as well, CMRs may not need to be executed *concomitantly*. Rather, preceding or subsequent movements of the nondominant hand, the foot, or even vocalizations may similarly facilitate the acquisition of multiple timing priors with the dominant hand. Notably, baseball batters typically initiate a leg kick before swinging a bat. Taken together, the present findings could contribute to extending Bayesian frameworks for understanding and improving daily sensorimotor behaviors.

### Limitations of the study

The potential neural bases underlying multiple-prior acquisition in timing were discussed. Although the SMA hypothesis is consistent with existing psychophysical^7^^,^^8^^,^^9^ and neurophysiological^17^^,^^18^^,^^19^^,^^20^^,^^21^^,^^22^^,^^23^^,^^24^ evidence, direct neurophysiological data are still lacking. Moreover, we discussed the potential effector-independent and effector-specific neural substrates for CMR effects, based on previous neurophysiological or neuroimaging studies on reaching-type movements^25^^,^^26^^,^^27^^,^^28^ and interlimb temporal coordination^30^^,^^31^^,^^32^^,^^33^; however, these inferences remain speculative. Future studies using neuroimaging or electrophysiological methods are needed to directly test such hypotheses and inferences.

Potential positive and negative effects of cognitive factors on multiple-prior acquisition were also discussed to interpret the difference in prior acquisition speed between the previous^9^ and current studies on timing. Based on previous findings on reaching^14^^,^^16^ and tactile temporal order judgments,^15^ we inferred that spatial cues and random prior ordering might have accelerated multiple-prior acquisition in the previous timing study, whereas color cues might have decelerated it in the present study. However, which of these factors is actually effective remains unclear at this stage. Identifying the effective factor is essential for determining the conditions that most strongly facilitate the acquisition of multiple timing priors and applying this knowledge to daily sensorimotor behaviors.

Task differences (reaching versus timing) likely explain the discrepancy in CMR effects with a foot response between the previous reaching study^10^ and the present timing study. Other methodological differences, such as the nature of the target variables (force fields versus statistical distributions), may also have contributed. Investigating CMR effects on statistical learning in reaching-type movements^1^^,^^47^ can directly test this possibility and clarify the extent to which CMRs contribute to multiple-prior acquisition across motor domains.

## Resource availability

### Lead contact

Requests for further information and resources should be directed to and will be fulfilled by the lead contact, Makoto Miyazaki (miyazaki-makoto@inf.shizuoka.ac.jp).

### Materials availability

This study did not generate new unique reagents.

### Data and code availability

• Data: All data to reproduce the results of this study are provided in the supplemental information (Data S1).
• Code: All original code used for data analysis is also provided in the supplemental information (Data S1).
• Additional information: Any additional information required to reanalyze the data reported in this paper is available from the lead contact upon request.

## Acknowledgments

This study was supported by 10.13039/501100001691JSPS 10.13039/501100001691KAKENHI (grant nos 25H01096, 22H00502, and 22K18263). The authors thank J. Ikki and K. Kimura for technical assistance with the experiments and N. Enomoto for administrative assistance with the experiments.

## Author contributions

Conceptualization, Y.T., R.T., N.W.R., and M.M.; data curation, Y.T., R.T., and M.M.; formal analysis, Y.T., R.T., and M.M.; investigation, Y.T. and R.T.; funding acquisition, M.M.; methodology, Y.T., R.T., N.W.R., and M.M.; project administration, M.M.; resources, M.M.; software, Y.T., R.T., N.W.R., and M.M.; supervision, M.M.; validation; Y.T., R.T., N.W.R., and M.M.; visualization, M.M.; writing – original draft, Y.T., R.T., and M.M.; writing – review & editing, N.W.R. and M.M.

## Declaration of interests

The authors declare no competing interests. Y.T. is currently employed by DENSO Corporation; this employment is unrelated to the present study, and the company had no role in this work.

## Declaration of generative AI and AI-assisted technologies in the writing process

During the preparation of this manuscript, M.M. used DeepL (DeepL SE, Cologne, Germany) and ChatGPT (OpenAI, San Francisco, CA, USA) in order to improve the clarity and grammar of parts of the manuscript. After using these tools, M.M. reviewed and edited all AI-assisted content as needed and takes full responsibility for the content of the publication.

## STAR★Methods

### Key resources table

**Table:** 

REAGENT or RESOURCE	SOURCE	IDENTIFIER
**Deposited data**		

Experimental data	Supplemental information	N/A

**Experimental models: Organisms/strains**		

60 participants in total [age: mean ± standard deviation (min–max)] - 20 participants in Experiment 1, 20.3 ± 1.9 (18–24) years, 8 female, 3 left-handed - 20 participants in Experiment 2, 20.5 ± 3.6 (18–34) years, 7 female, 2 left-handed - 20 participants in Experiment 3, 20.2 ± 1.7 (18–23) years, 7 female, 2 left-handed	N/A	N/A

**Software and algorithms**		

MATLAB, R2025a	Mathworks	https://matlab.mathworks.com/
Code for model fitting and parameter estimation	Supplemental information	N/A

### Experimental model and study participant details

#### Participants

Sixty healthy individuals participated in Experiments 1–3 [age 20.3 ± 2.5 (18–34) years, mean ± standard deviation (SD) (min–max); 22 females]. Seven participants were left-handed. Participants were recruited via poster and online advertisements at universities in and around Hamamatsu City. Applicants were assigned to experiments in the order of application (20 participants per experiment; see the key resources table for participant demographics in each experiment). Age and sex were recorded for all participants. Sex-related effects were not analyzed, as the present study focused on general sensorimotor timing mechanisms rather than sex-specific differences, and the experiments were not designed to examine sex-related effects. Accordingly, potential sex-related influences on the results cannot be ruled out and remain an open question for future studies. Information on race, ethnicity, or ancestry was not collected because these variables were not relevant to the study objectives, which focused on general sensorimotor timing mechanisms, and were not included in the approved ethics protocol. Participants were sampled without overlap across experiments. All participants were naïve to the purpose of the experiments and had not performed similar timing tasks, to prevent potential effects of previous experience.

#### Ethics statement

This study was approved by the Ethics Committee of Shizuoka University (approval number: 15-19). All experiments were conducted in accordance with the approved guidelines and regulations. Written informed consent was obtained from all participants.

### Method details

#### Stimuli

In a dimly lit, sound-shielded room, each participant sat on a chair with their head positioned on a chin rest, 71 cm from the cathode-ray tube monitor (Eizo FlexScan T565, Japan; 100 Hz, 17 inch). Stimuli were generated, and participant responses were recorded using NBS Presentation (Neurobehavioral Systems, USA).

In each trial, three sequential stimuli (S1, S2, and S3; each duration: 110 ms) were presented at the center of the display (Figure 1A). Each stimulus sequence was colored either green or orange. The diameter of the circular frame in which the stimuli appeared was 1° of the visual angle.

In each trial, the *T*_*S*_ between S1 and S2 was identical to that between S2 and S3. *T*_*S*_ was randomly selected from one of two discrete prior distributions: the short prior (450, 600, 750, 900, and 1050 ms; *μ*_prior_ = 750 ms) or the long prior (1200, 1350, 1500, 1650, and 1800 ms; *μ*_prior_ = 1500 ms) (Figure 1B). In accordance with the procedures in previous studies,^3^^,^^4^^,^^7^^,^^48^^,^^49^ we used uniform distributions for the priors, which assist efficient data acquisition. Gaussian prior distributions result in fewer data for the shorter and longer *T*_*S*_, which can be avoided using uniform distributions. Moreover, even when a uniform distribution is used as the prior, participants acquire an internal Gaussian prior,^3^^,^^7^^,^^50^ allowing the application of the Bayesian model^1^^,^^2^ to our tasks. The two priors were assigned to either green or orange stimuli, which alternated from trial to trial (Figure 1C).

#### Tasks

In all experiments, participants attempted to press a button using their dominant index finger at the moment S3 appeared, based on the *T*_*S*_ between S1 and S2 as a reference (coincidence timing task). During the task, they fixated on the frame circle where the stimuli were presented. No feedback was given regarding the accuracy of their timing response.

In Experiment 1 (Figure 3A), participants performed the timing task using only their dominant index finger regardless of the stimulus color (i.e., prior). In Experiment 2 (Figure 4A), participants pressed an additional button with their nondominant hand along with the dominant-hand timing response, selectively for one of the stimulus colors (i.e., prior). In Experiment 3 (Figure 5A), participants pressed a foot switch with the contralateral forefoot along with the dominant-hand timing response, selectively for one of the stimulus colors (i.e., prior). Half of the participants performed the additional nondominant hand response (Experiment 2) or contralateral foot response (Experiment 3) when green stimuli were presented; the remaining participants did so for orange stimuli. All combinations of conditions (priors, stimulus colors, and presence or absence of CMR) were counterbalanced across participants.

The lateral (left-right) distances between the centers of the two hand buttons in Experiment 2, and between those of the hand button and foot switch in Experiment 3, were 11.5 cm. Hand and foot responses were recorded using a USB response pad with a customized button layout and a foot switch pedal (Black Box Toolkit Ltd., UK).

#### Procedure

Each participant completed 640 trials of the timing task (40 trials per session × 16 sessions). The interval between the onset of S3 and that of S1 in the following trial was 3.1 s. A short beep (0.2 s) was presented 1 s before S1 to indicate the start of the trial. Participants took a 1-min rest after each session and a 5-min rest after every four sessions. The rest periods were extended if participants reported fatigue or drowsiness.

#### Data analysis

We measured *T*_*R*_ for each trial (Figure 1A). Trials were excluded if they contained erroneous responses or if the same button(s) or foot switch were pressed two or more times within a trial. We also excluded *T*_*R*_ values that lay outside the range of the mean ± 3 × SD for each *T*_*S*_ within each trial bin (320 trials per bin) for each participant. For all analyses, we used the *T*_*R*_ values from the dominant hand. The onset of CMRs occurred a few milliseconds or a few tens of milliseconds later than the responses by the dominant hand. In Experiment 2, this delay was 3.81 ± 7.20 ms (mean ± SD across participants) (*t*_19_ = 2.36, *p* = 0.029, Pearson’s *r* = 0.48, two-tailed paired *t*-test). In Experiment 3, the delay was 21.34 ± 16.30 ms (*t*_19_ = 5.86, *p* = 1.2 × 10^-5^, *r* = 0.80).

Based on the Bayesian estimation model of the coincidence timing task,^8^^,^^9^ the mean response time interval across trials, ${\bar{T}}_{R}$, is expressed as:

(Equation 1) $${\bar{T}}_{R}=\frac{\sigma_{\text{prior}}^{2}}{\sigma_{\text{prior}}^{2}+w^{2}{T_{S}}^{2}}T_{S}+\frac{w^{2}{T_{S}}^{2}}{\sigma_{\text{prior}}^{2}+w^{2}{T_{S}}^{2}}\mu_{\text{prior}}$$

where *σ*_prior_ denotes the SD of the prior distribution. *w* denotes the Weber fraction, which accounts for scalar variability^11^^,^^51^ as follows:

(Equation 2) $$\sigma_{\text{sensed}}=wT_{S}$$

where *σ*_sensed_ denotes the SD of the sensed *T*_*S*_. Equation 2 indicates that sensory uncertainty increases as *T*_*S*_ becomes longer.

According to Equation 1, the ${\bar{T}}_{R}$ × *T*_*S*_ function forms a curve with a shallower gradient at longer *T*_*S*_ (Figure 2A). This reflects increased reliance on *μ*_prior_ when sensory uncertainty is greater. Equation 1 yields the following predictions for experimental outcomes. If participants acquired one generalized prior, the curves corresponding to short and long priors would overlap (left panel, Figure 2A). Conversely, if participants acquired two independent priors, two distinct curves would appear (right panel, Figure 2A).

For each participant, we averaged *T*_*R*_ values across every 320 trials (160 trials per prior) to compute the ${\bar{T}}_{R}$ values in each *T*_*S*_. Subsequently, based on Equation 3, we fitted curves to the ${\bar{T}}_{R}$ values as a function of *T*_*S*_ using the least-squares method.

(Equation 3) $${\bar{T}}_{R}=\left\{ \begin{array}{c} \frac{\sigma_{\text{short}}^{2}}{\sigma_{\text{short}}^{2}+w^{2}{T_{S}}^{2}}T_{S}+\frac{w^{2}{T_{S}}^{2}}{\sigma_{\text{short}}^{2}+w^{2}{T_{S}}^{2}}\mu_{\text{short}\;}\;\text{for}\;450\;\text{ms}\leq T_{S}\leq 1050\;\text{ms}\;\\ \frac{\sigma_{\text{long}}^{2}}{\sigma_{\text{long}}^{2}+w^{2}{T_{S}}^{2}}T_{S}+\frac{w^{2}{T_{S}}^{2}}{\sigma_{\text{long}}^{2}+w^{2}{T_{S}}^{2}}\mu_{\text{long}}\;\text{for}\;1200\;\text{ms}\leq T_{S}\leq 1800\;\text{ms}\; \end{array}\right.$$

where *μ*_short_ and *μ*_long_ denote the means of the short and long prior distributions, respectively. *σ*_short_ and *σ*_long_ denote SDs of the short and long prior distributions, respectively. *w* is shared by the two priors. As initial values, we set *μ*_short_ to 750 ms and *μ*_long_ to 1500 ms, and both *σ*_short_ and *σ*_long_ to 237 ms. These values corresponded to the true parameters of the prior distributions used in the experiments. The initial value of *w* was set to 0.15 based on a previous study.^3^

To evaluate whether participants acquired two independent priors, we derived ${\bar{T}}_{R}\left({Μ}_{\text{priors}}\right)$ values (Figure 2B) from the fitted curves for each participant. If participants acquired one generalized prior, the ${\bar{T}}_{R}\left({Μ}_{\text{priors}}\right)$ values would not differ between the two priors. Conversely, if participants acquired two independent priors, ${\bar{T}}_{R}\left({Μ}_{\text{priors}}\right)$ values would be higher for the long prior than for the short prior.

In theory, we can infer ${\hat{\mu }}_{\text{prior}}$ (*μ*_short_ and *μ*_long_ in Equation 3) from the point at which the ${\bar{T}}_{R}$ × *T*_*S*_ curve intersects the unity line (Figure 2C). However, in practice, the intersection points are sensitive to idiosyncratic responses, such as overshoots, undershoots, or slopes steeper than unity. The ${\hat{\mu }}_{\text{prior}}$ values were either less than the minimum *T*_*S*_ (450 ms) or greater than the maximum *T*_*S*_ (1800 ms) in 32.5% of curve fittings in Experiment 1, 37.5% in Experiment 2, and 15.0% in Experiment 3. Some of these values were highly implausible (e.g., < −10000 ms, > 10000 ms). To address this issue, we calculated ${\hat{\mu }}_{\text{prior}}$ using grand-averaged ${\bar{T}}_{R}$ values, which can average out individual idiosyncratic responses.^8^^,^^9^

However, in Experiment 2, the ${\hat{\mu }}_{\text{prior}}$ for the short prior exhibited irregular values (−50635.5 ms in trials 1–320, −36617.5 ms in trials 321–640), even when using the grand-averaged ${\bar{T}}_{R}$ values. This irregularity was due to systematic undershoots (i.e., shorter intervals) in ${\bar{T}}_{R}$. To calculate the ${\hat{\mu }}_{\text{prior}}$ values in Experiment 2, based on the method used by Cicchini et al.,^3^ we compensated for systematic undershoots before curve fitting. First, we calculated the systematic undershoot by averaging the difference between the grand-averaged ${\bar{T}}_{R}$ values and the corresponding *T*_*S*_ (−48.4 ms in trials 1–320, −54.3 ms in trials 321–640). Next, we subtracted this difference from the original ${\bar{T}}_{R}$ values for each *T*_*S*_. We then conducted curve fittings. This yielded ${\hat{\mu }}_{\text{prior}}$ values for the short prior that fell within plausible ranges (1224.0 ms in trials 1–320, 1129.2 ms in trials 321–640). In addition, ${\hat{\mu }}_{\text{prior}}$ values shown in Figure 4D were re-adjusted based on the differences in the ${\hat{\mu }}_{\text{prior}}$ values for the long prior, calculated before and after the subtraction of the systematic bias (218.5 ms in trials 1–320, 238.6 ms in trials 321–640, where original < subtracted). Consequently, the ${\hat{\mu }}_{\text{prior}}$ values exhibited consistent profiles with the ${\bar{T}}_{R}\left({Μ}_{\text{priors}}\right)$ values (Figure 4C).

Notably, the ${\hat{\mu }}_{\text{prior}}$ and ${\bar{T}}_{R}\left({Μ}_{\text{priors}}\right)$ values are uniquely determined by the model fitting regardless of the initial values. However, the *σ*_short_, *σ*_long_, and *w* values vary depending on the initial values. The *σ*_short_, *σ*_long_, and *w* values obtained from the fittings are shown in Table S1.

### Quantification and statistical analysis

To test whether participants acquired two independent priors in each experiment, we performed a repeated-measures two-way ANOVA with factors of prior and trial bin on the ${\bar{T}}_{R}\left({Μ}_{\text{priors}}\right)$ values. The normality of the residuals and the homogeneity of variances across priors and trial bins were not rejected (normality: Experiment 1, *W* = 0.99, *p* = 0.48; Experiment 2, *W* = 0.98, *p* = 0.45; Experiment 3, *W* = 0.99, *p* = 0.86; Shapiro–Wilk test; homogeneity: Experiment 1, *χ*^2^_3_ = 6.49, *p* = 0.090; Experiment 2, *χ*^2^_3_ = 4.40, *p* = 0.22; Experiment 3, *χ*^2^_3_ = 0.046, *p* = 0.998; Bartlett’s test), supporting the use of the ANOVAs.

To assess CMR effects on the acquisition of two priors, we tested whether the $\Delta {\bar{T}}_{R}\left({Μ}_{\text{priors}}\right)$ values in Experiments 2 and 3 were greater than those observed in the control experiment without CMR (Experiment 1). The $\Delta {\bar{T}}_{R}\left({Μ}_{\text{priors}}\right)$ values were obtained by subtracting the ${\bar{T}}_{R}\left({Μ}_{\text{priors}}\right)$ values for the short prior from those for the long prior in each trial bin for each experiment. Such a subtraction procedure can isolate the factor or interaction of interest.^52^ We conducted a mixed-design two-way ANOVA on the $\Delta {\bar{T}}_{R}\left({Μ}_{\text{priors}}\right)$ values, with one between-participant factor of experiment and one within-participant factor of trial bin. The normality of the residuals and the homogeneity of variances across experiments and trial bins were not rejected (normality: *W* = 0.98, *p* = 0.19; homogeneity: *χ*^2^_5_ = 3.80, *p* = 0.58), thus supporting the ANOVAs.

For post-hoc simple effect analyses, the significance threshold *α* was corrected using the Bonferroni method for multiple comparisons. We used *η*_*p*_^2^ as the effect size index for the ANOVAs.

To further statistically test the CMR effects on $\Delta {\hat{\mu }}_{\text{prior}}$ values in Experiments 2 and 3, we applied the permutation method, using those in Experiment 1 as the control. We first randomly permuted the individuals’ ${\bar{T}}_{R}$ sequences against *T*_*S*_ for each trial bin across the two experiments (Experiment 2–control or Experiment 3–control). We then averaged the permuted ${\bar{T}}_{R}$ sequences across the 20 individuals in each experiment. For each trial bin in each experiment, we generated 10000 sets of grand-averaged permutation ${\bar{T}}_{R}$ sequences (40000 sets in total) and performed curve fittings on them to obtain the ${\hat{\mu }}_{\text{prior}}$ values. We next calculated the differences in the permutation $\Delta {\hat{\mu }}_{\text{prior}}$ values between the experiments (Experiment 2 > control or Experiment 3 > control). We counted how many of these permutation differences exceeded the original difference (*n*_perm > org_) and computed the permutation *p*-value (*n*_perm > org_/10000) for each trial bin, applying a Bonferroni correction.

For the permutation analyses between Experiment 2 and the control, to eliminate the influence of systematic undershoots in Experiment 2, we performed curve fittings on the grand-averaged permutation ${\bar{T}}_{R}$ sequences after subtracting the systematic biases. As a result, no irregular ${\hat{\mu }}_{\text{prior}}$ values were found in the permutation data. In the permutation analyses between Experiment 3 and the control, irregular ${\hat{\mu }}_{\text{prior}}$ values were observed for the short prior in 0.065% of the permutation data. We removed these irregular values before conducting the subsequent analyses. Even when calculating the permutation *p*-values without removing the irregular ${\hat{\mu }}_{\text{prior}}$ values, we reached the same statistical conclusions (trials 1–320: permutation *p* = 0.28 > 0.05/2; trials 321–640: permutation *p* = 0.0049 < 0.05/2).

Statistical significance in ANOVAs (Figures 3, 4, and 5) and permutation analyses (Figures 4 and 5) is indicated by asterisks (∗ *p* < 0.05/2, ∗∗ *p* < 0.01/2, ∗∗∗ *p* < 0.001/2, Bonferroni corrected).

In addition, based on the Akaike information criterion (AIC),^53^ we further evaluated whether the two-prior model (Equation 3) provided a better fit than the one-prior model (Equation 4) to explain the ${\bar{T}}_{R}$ × *T*_*S*_ functions.

(Equation 4) $${\bar{T}}_{R}=\frac{\sigma_{\text{wide}}^{2}}{\sigma_{\text{wide}}^{2}+w^{2}{T_{S}}^{2}}T_{S}+\frac{w^{2}{T_{S}}^{2}}{\sigma_{\text{wide}}^{2}+w^{2}{T_{S}}^{2}}\mu_{\text{wide}}\;\text{for}\;450\;\text{ms}\leq T_{S}\leq 1800\;\text{ms}$$

where *μ*_wide_ and *σ*_wide_ denote the mean and SD of the generalized prior distribution, respectively. The one-prior model assumes the acquisition of one generalized prior, whereas the two-prior model assumes the acquisition of two independent priors. First, we fitted both the one-prior and two-prior models to the grand-averaged ${\bar{T}}_{R}$ values. For fitting the one-prior model, the initial values of *μ*_wide_, *σ*_wide_, and *w* were set to 1125 ms, 454 ms, and 0.15*,* respectively. Next, we computed AIC corrected for small samples (AIC*c*) using the residuals of ${\bar{T}}_{R}$ values for each participant from each fitted curve. Using the AIC*c* values, we calculated Akaike weights^12^ for the one-prior and two-prior models. Higher Akaike weights for the two-prior model indicate stronger evidence for the acquisition of two independent priors. The AIC*c* values are shown in Table S2.

## Supplemental information

Document S1. Tables S1–S2

Data S1. This zipped file includes the data and analysis code to reproduce the results reported in this study, together with a README file
