# Supplementary material for: Concomitant motor responses facilitate the acquisition of multiple timing priors beyond upper-limb contexts
Source: iScience. 2026 Feb 17;29(3):115051. doi: 10.1016/j.isci.2026.115051 (PMC12992963; doi:10.1016/j.isci.2026.115051)
Supplement: Document S1. Tables S1–S2 [file mmc1.pdf]

**iScience, Volume *29***

## Supplemental information

# Concomitant motor responses facilitate the acquisition of multiple timing priors beyond upper-limb contexts

**Yuma Tanaka, Riku Takaki, Neil W. Roach, and Makoto Miyazaki**

**Table S1.** $\sigma_{short}$, $\sigma_{long}$ (unit: ms), and $w$ values across participants [median (first/third quartile)] obtained from the fittings per 320 trials in each experiment.

| | Trial | $\sigma_{short}$ | $\sigma_{long}$ | $w$ |
|---|---|---|---|---|
| Experiment 1 | 1–320 | 644.6 (375.4/929.7) | 253.0 (104.6/350.5) | 0.085 (0.017/0.14) |
| | 321–640 | 798.4 (326.7/1060.4) | 204.0 (90.2/330.3) | 0.064 (0.020/0.13) |
| Experiment 2 | 1–320 | 797.2 (408.1/911.2) | 91.9 (49.0/316.8) | 0.049 (0.016/0.13) |
| | 321–640 | 593.8 (416.6/794.0) | 225.1 (91.9/320.1) | 0.10 (0.020/0.12) |
| Experiment 3 | 1–320 | 313.8 (269.5/423.7) | 319.5 (248.2/347.5) | 0.13 (0.12/0.15) |
| | 321–640 | 329.3 (251.6/520.3) | 294.9 (234.3/382.6) | 0.14 (0.12/0.15) |

**Table S2.** Akaike information criterion corrected for small samples (AIC*c*) values for the one-prior and two-prior models, calculated per 320 trials in each experiment. Smaller AIC*c* values indicate better fits.

| | Trial | **One-prior** | **Two-prior** |
|---|---|---|---|
| Experiment 1 | 1–320 | 1621.2 | 1617.7 |
| | 321–640 | 1611.5 | 1612.9 |
| Experiment 2 | 1–320 | 1606.0 | 1606.8 |
| | 321–640 | 1591.6 | 1583.5 |
| Experiment 3 | 1–320 | 1615.6 | 1613.6 |
| | 321–640 | 1683.3 | 1670.8 |
