# Supplementary material for: Concomitant motor responses facilitate the acquisition of multiple timing priors beyond upper-limb contexts
Source: iScience. 2026 Feb 17;29(3):115051. doi: 10.1016/j.isci.2026.115051 (PMC12992963; doi:10.1016/j.isci.2026.115051)
Supplement: Data S1. This zipped file includes the data and analysis code to reproduce the results reported in this study, together with a README file [file mmc2.zip › Data S1/README_data_code_files.pdf]

List of data and code files and their contents

(1) 'Data_Exp1.xlsx', 'Data_Exp2.xlsx', 'Data_Exp3.xlsx'

These files contain raw data for Experiments 1, 2, and 3, respectively. In each file, sheets '1' to '20' contain the trial-by-trial stimuli and responses for each participant. The sheet names correspond to the anonymized participant numbers.

(2) 'Results_Exp1-3.xlsx'

This file contains the analyzed data for all experiments. The sheet labelled 'Each participant' contains the $\bar{T}_R$ values as a function of T_S (Figures 3B [Exp. 1], 4B [Exp. 2], and 5B [Exp. 3]) and the results of the model fittings for each participant (Figures 3C [Exp. 1], 4C and 4E [Exp. 2], 5C and 5E [Exp. 3], and Table S1). The sheet labelled 'Grand average' contains the grand averages (i.e., means across participants) of the $\bar{T}_R$ values and the results derived from them (Figures 3D [Exp. 1], 4D [Exp. 2], 5D [Exp. 3], and Table S2). The sheet labelled 'Permutation' contains the results of the permutation analyses (Figures 4F [Exp. 2] and 5F [Exp. 3]).

(3) 'Code1_sort_fit_eachparticipant.m'

This code sorts the T_R values for each participant in files (1) as a function of T_S. It then computes the mean T_R ($\bar{T}_R$) values for each T_S and fits the two-prior model (Equation 3) to the resulting $\bar{T}_R$ values. The obtained results are recorded in the 'Each participant' sheet of file (2) (already recorded). MATLAB with the Optimization Toolbox is required to run this and the subsequent codes.

(4) 'Code2_fit_AIC_grandaverage.m'

This code computes the grand-averaged $\bar{T}_R$ values and fits the two-prior model to them. It also computes the AIC*c* values and Akaike weights using the residuals of the $\bar{T}_R$ values for each participant relative to the curves fitted to the grand-averaged $\bar{T}_R$ values. The obtained results are recorded in the 'Grand average' sheet of file (2) (already recorded). This code should be run after Code1 has finished.

(5) 'Code3_permutation.m'

This code generates the permutation data and computes the permutation p-values for the differences in $\Delta\hat{\mu}_{\text{prior}}$ between the two experiments (Exp. 2 > Exp. 1 or Exp. 3 > Exp. 1). The obtained results are recorded in the 'Permutation' sheet of file (2) (already recorded). This code should be run after Code2 has finished.

Depending on the software version or hardware environment, Codes 1–3 may produce minute numerical differences below significant digits. The results recorded in file (2) were computed using MATLAB R2025a on Windows 11 Pro on a personal computer with an Intel® Core™ Ultra Processor 255H (CF-SC6BRVCP, Let's Note SC, Panasonic, Japan).

Abbreviations used in the data and code files

TS, Stimulus time interval (T_S)

TR, Response time interval (T_R)

mTR, Mean T_R across trials ($\bar{T}_R$)

mTRM, $\bar{T}_R$ at $T_S = M_{\text{priors}}$ [$\bar{T}_R(M_{\text{priors}})$], where M_{priors} denotes the mean over the two prior distributions.

mp, Mean of the acquired prior distribution ($\hat{\mu}_{\text{prior}}$)

dmp, Divergence in $\hat{\mu}_{\text{prior}}$ between the two priors ($\Delta\hat{\mu}_{\text{prior}}$)

ddmp, Difference in $\Delta\hat{\mu}_{\text{prior}}$ between the experiments

sdp, Standard deviation of the prior distribution (σ_{prior})

w, Weber fraction
